# Anticoagulants Interfere With the Angiogenic and Regenerative Responses Mediated by Platelets

**DOI:** 10.3389/fbioe.2020.00223

**Published:** 2020-03-20

**Authors:** Paula Oneto, Paula Romina Zubiry, Mirta Schattner, Julia Etulain

**Affiliations:** Laboratory of Experimental Thrombosis, Institute of Experimental Medicine, CONICET-National Academy of Medicine, Buenos Aires, Argentina

**Keywords:** acid-citrate-dextrose, angiogenesis, anticoagulants, glucose, platelet-rich-plasma, trisodium-citrate, regeneration

## Abstract

**Background and aims:**

Platelet rich plasma (PRP) obtained from blood anticoagulated with acid-citrate-dextrose (ACD) or sodium-citrate (SC) is used for regenerative medicine as source of platelet-derived growth factors. Allergic reactions against citrate were reported in patients after local injection of PRP allowing us to hypothesize that anticoagulants exert a harmful and local effect that interferes with the regenerative proprieties of platelets. Herein we test this hypothesis by analyzing the effect of ACD and SC on angiogenic and regenerative responses mediated by platelets.

**Methods:**

PRP was obtained from SC- or ACD-anticoagulated blood; platelets were lysed to release growth factors; and PRP releasates (PRPr) were used to induce *in vitro* endothelial proliferation and 2D-migration, and regeneration of mouse skin wounds.

**Results:**

We first compared proliferation and migration of endothelial cells mediated by anticoagulated-PRPr supplemented or not with CaCl_2_. Alteration of endothelial adhesion and impediment of proliferation and migration was observed without CaCl_2_. Although endothelial morphology was normalized in SC- and ACD-PRPr after calcium restitution, angiogenic responses were only markedly induced by SC-PRPr. *In vivo* studies revealed a delay in mouse skin regeneration after treatment with anticoagulated-PRPr without CaCl_2_. Healing was only induced after calcium restitution in SC- but ACD-PRPr. Moreover, the development of inflammatory intradermal papules was evidenced after injection of ACD-PRPr. Supplementation of SC-PRPr with the equivalent concentration of dextrose (D-Glucose, 18 mM) present in ACD-PRPr resulted in reduction of endothelial proliferation and migration, delay of mouse skin regeneration and development of intradermal papules. Finally, collecting blood with half amount of SC significantly improved all the angiogenic and regenerative responses mediated by PRPr. In contrast, the delay of skin regeneration and the development of inflammatory papules remained stable after dilution of ACD.

**Conclusion:**

Our findings indicate that (1) calcium restitution is required to impair the cellular and tissue alterations induced by citrated-anticoagulants contained in PRP; (2) ACD-derived dextrose confers anti-angiogenic, anti-regenerative and pro-inflammatory proprieties to PRP; and (3) half concentration of SC improves the angiogenesis and regeneration mediated by PRP.

## Introduction

Besides playing a key role in thrombosis and hemostasis platelets also promote tissue regeneration due to the action of several growth factors and cytokines released from their granules. For this reason, platelet-rich plasma (PRP) based therapies are used in regenerative medicine for treating several clinical conditions including ulcers, burns, muscle damage, bone diseases and in tissue recovery after surgery (Etulain, 2018; Anitua et al., 2019). Despite the large variability of applications, the efficacy of regenerative treatments using PRP has been called into question due to the absence of large controlled clinical trials and consensus regarding PRP preparation techniques. The procedures currently used for this purpose are derived from classical protocols for obtaining platelet concentrates for transfusion, coagulation assays or platelet functionality, which are focused on preserving platelet hemostatic responses (Etulain et al., 2018). Most of these protocols use citrate as a common element in anticoagulant-conservative solutions. Citrate-based anticoagulants prevent coagulation by their ability to chelate ionized calcium present in the blood to form non-ionized calcium-citrate complexes (Mariano et al., 2011). This allows to preserved the hemostatic functionality of platelets for long periods of time (5–7 days) and it is also safe and efficient for transfusions since citrate is diluted in volemia and rapidly metabolized to bicarbonate by the tricarboxylic pathway in liver, kidney, and skeletal muscle (Leparc, 1985; Mariano et al., 2011). Based on these classical settings, acid-citrate-dextrose (ACD) and sodium citrate (SC) solutions are commonly used for collecting blood in the context of regenerative protocols mediated by platelets (The International Cellular Medical Society, 2012; Dhurat and Sukesh, 2014; Anitua et al., 2016). The studies about the effect of anticoagulants in the context of regenerative treatments with platelets are only focused on optimizing platelets number, coagulation or platelet-derived growth factors levels (O’Neill et al., 2001; Giraldo et al., 2015; do Amaral et al., 2016; Gonzalez et al., 2016; Singh, 2018) but they do not analyze the functionality of platelets on target tissue neither the possible adverse consequences caused by anticoagulants additives in platelet solutions. Notably, adverse effects have been reported in patients in the context of tendinopathies treatments with PRP including swelling, erythema, edema, and pain (Bowman et al., 2013; Kaux et al., 2014); skin redness, swelling, bruising, pruritus, scaling, and dryness after intra-dermal injection of PRP in the context of skin rejuvenation (Alam et al., 2018); and the systemic allergic reaction post-injection of PRP into a bone cyst (Latalski et al., 2019). Although the potential mediators/mechanisms involved in these effects were not addressed, one of these studies revealed the intradermal allergic reaction against citrate during skin allergic test (Latalski et al., 2019) suggesting a possible toxic effect caused by the anticoagulant additive in PRP. To further understand the local effect of citrate on tissue regeneration we evaluated the effect of ACD and SC analyzing their effect on the target phenomena of angiogenesis and regeneration mediated by platelets.

## Materials and Methods

### Ethics Statements

Human blood studies were conducted according with the Declaration of Helsinki and the Ethic Committee of the National Academy of Medicine (T.I. 12910/18/X). All subjects provided informed written consent for the collection of samples and subsequent analysis.

Animal studies received the approval of the Institutional Committee for Care and Use of Laboratory Animals (CICUAL 059/2018) and were conducted according to principles set forth in the *Guide for the Care and Use of Laboratory Animals* (8th Ed. NRC, United States) and by the European Parliament and Council concerning the protection of animals used for scientific purposes (Directive 2010/63/EU).

### Preparation of PRP Releasates (PRPr)

Blood samples were obtained by venous puncture from healthy donors that assist to the Hemotherapy Service of Hospital Fernández (CABA, Argentina), and who had not taken non-steroidal anti-inflammatory drugs during the 10 days before sampling. Blood was collected in tubes containing trisodium citrate (SC) 3.8% (1:9 v/v) or ACD (Formula A solution, trisodium citrate 22.0 g/L, citric acid 7.3 g/L, and dextrose 21.8 g/L, 1.5:8.5 v/v).

Blood samples were store at room temperature for a maximum of 40 min before PRP preparation. Blood was centrifuged at 180 × *g* for 10 min to obtain PRP and platelets were lysed by two cycles of freezing and thawing (−80°C, 15 min). After PRP separation, remaining blood was centrifuged at 890 × *g* for 10 min to obtain platelet poor plasma (PPP). Then, coagulation in lysed PRP and PPP was induced by adding CaCl_2_ (22–25 mM). After 40 min of incubation at 37°C, clot was removed and the PRP releasates (PRPr) and PPP were centrifuged at 890 × *g* for 10 min and then stored at −80°C until used.

### *In vitro* Endothelial Proliferation and 2D-Migration Assays

Human microvascular endothelial cells (HMEC-1) were grown in MCDB-131 supplemented with fetal bovine serum (FBS) (10%), L-glutamine (10 mM) and streptomycin (100 μg/mL), penicillin (100 U/mL), EGF (10 ng/mL), and hydrocortisone (1 μg/mL) at 37°C in a humidified 5% CO_2_ incubator (Etulain et al., 2018). Human umbilical vein endothelial cells (HUVEC) were obtained from four different donors as previously described (Mena et al., 2018) and were expanded and maintained in Endothelial Growth Medium 2 (EGM2; Lonza, Walkersville, MD, United States) containing Endothelial Basal Medium 2 (EBM2) supplemented with FBS, hydrocortisone, human fibroblast growth factor (hFGF), VEGF, human long R3, insulin-like growth factor-1 (R3-IGF-1), ascorbic acid, human epidermal growth factor (hEGF), gentamicin, amphotericin-B and heparin (Mena et al., 2014). After washing, growth factor-enriched medium was totally replaced by PRPr. The proliferation of endothelial cells (15 × 10^3^, indicated as dotted line in Figures) was determined at 24 h by measuring acid phosphatase activity and confirmed by Neubauer chamber cell counting. Migration into scratched confluent monolayers of endothelial cells was analyzed at 8 h with ImageJ software and the percentage of migration was calculated as [(wound area at 0 h - wound area at 8 h)/wound area at 0 h] × 100 (Etulain et al., 2013, 2018). Evaluation of endothelial viability was analyzed under optical microscopy by trypan blue exclusion test.

### Growth Factors Determination

Levels of VEGF (RayBiotech, Inc., Norcross, GA, United States), bFGF (Biolegend, San Diego, CA, United States), EGF (Life Technologies, Carlsbad, CA, United States), PDGF (Abcam, Cambridge, United Kingdom), and TGFβ (Biolegend, San Diego, CA, United States) in PRPr were determined by ELISA.

### Mice

Female BALB/c mice, 8–10 weeks old, originally purchased from Charles River Research Animal Diagnostic Services (Wilmington, MA, United States) were housed in a controlled environment with free access to water and a standard diet in the animal facility of the Institute of Experimental Medicine (Etulain et al., 2018).

### Model of Wound Healing in Mice

Platelet rich plasma was obtained from orbital blood of anesthetized mice and collected in SC or ACD. Platelets were lysed at −80°C during 15 min, and releasates were obtained after centrifugation. PRPr were supplemented or not with CaCl_2_ immediately before being injected subcutaneously in the periphery of four round full-thickness excisional wounds of 3 mm diameter generated in the back skin of other anesthetized mice (Etulain et al., 2018). Saline (0.9 g NaCl/100 ml distilled water) was used as control. Wound healing was analyzed at 3 and 6 days post-injury. Back skin of mice was photographed and the perimeter of the wound area was calculated using the ImageJ software. Results were expressed as a percentage of the area at day 0 when wounds were excised. The presence of intradermal infiltrates (papules) in the periphery of wounds was quantified macroscopically. Anesthetized mice were culled, and paraffin sections of skin wounds were stained with Masson’s trichrome. Images were captured using an inverted microscope and analyzed using the ImageJ software.

### Statistical Analysis

Results are expressed as means ± SEM. A *P* < 0.05 was considered statistically significant. The Shapiro-Wilk test was used to define state normality and equal variance. In case of sample normality and equal variance, parametric tests such us one- or two-way analysis of variance (ANOVA) followed by Fisher’s test were used. If normality assumption failed, non-parametric such us Kruskal Wallis follows by Dunns or Mann-Whitney tests were performed. All the tests were used according to the experimental design and analyzed with GraphPad software (PRISM Version 8.0, San Diego, CA, United States).

## Results

### The Reduction of Calcium Levels Exerted by Anticoagulants Interferes With Endothelial Proliferation and Migration Mediated by PRPr *in vitro*

**FIGURE 1:**
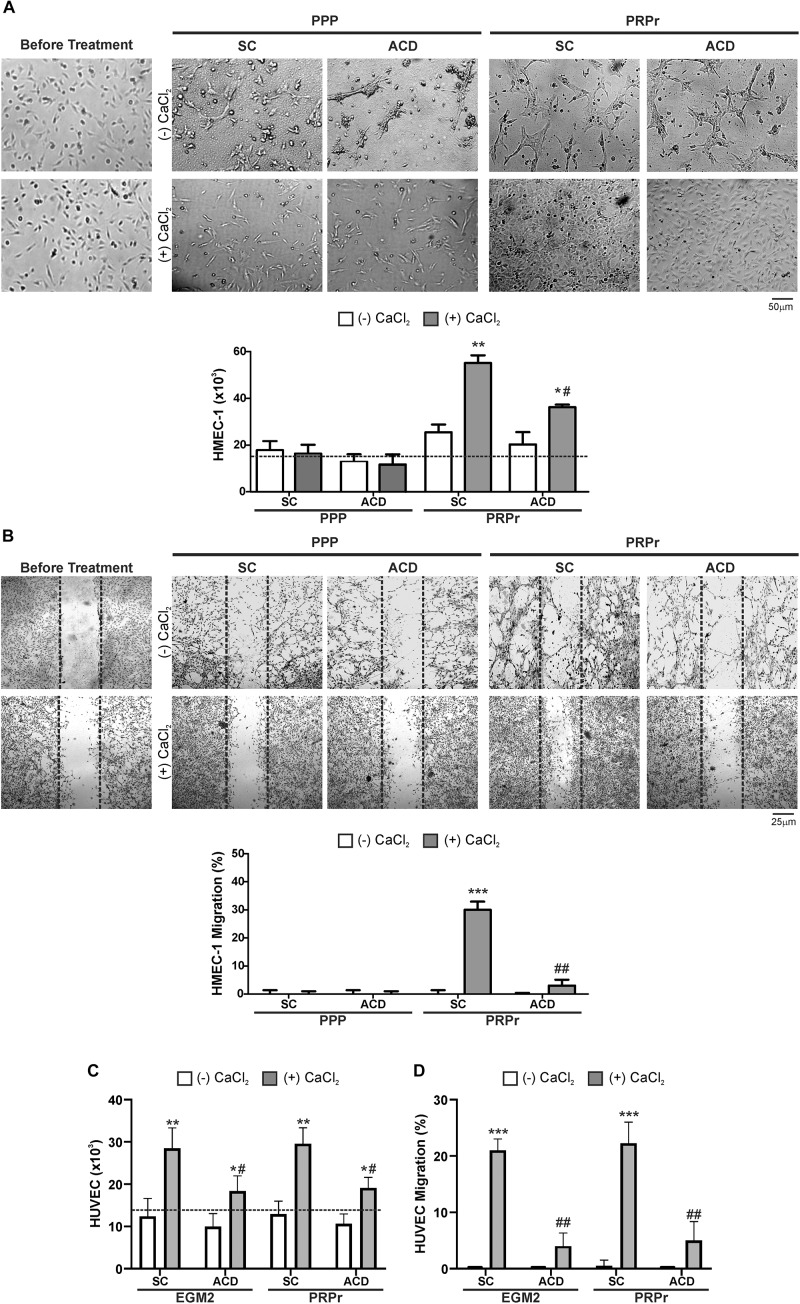
The effect of calcium restitution and citrate-anticoagulants in endothelial proliferation and migration mediated by PRPr. **(A,B)** Platelets contained in SC-PRP and ACD- were lysed to release total amount of platelet-derived growth factors and then coagulated or not with CaCl_2_. Clot was removed and PRPr releasates (PRPr) were incubated with HMEC-1. SC-PPP and ACD- coagulated or not with CaCl_2_ were used as control. Endothelial cell proliferation at 24 h **(A)** and migration at 8 h **(B)** were determined. Images are representative of seven independent experiments and were obtained before and after treatments (magnification 40X). **(C–D)** HUVEC were incubated with SC-PRPr and ACD- supplemented or not with CaCl_2_. SC-EGM2 or ACD- supplemented or not with CaCl_2_ was used as control. Endothelial proliferation at 24 h **(C)** and migration at 8 h **(D)** were determined (*n* = 4–7; *P < 0.05, **P < 0.01, ***P < 0.001 vs. CaCl_2_ (-); ^#^*P* < 0.05, ^##^*P* < 0.01 vs. SC-PRPr or SC-EGM2. Two-way ANOVA followed by Fisher test).

Considering that calcium is a second messenger essential for the cellular homeostasis (Dubois et al., 2016; Bootman et al., 2018) we first evaluated whether anticoagulants interferes with angiogenic responses mediated by platelets by chelating calcium. To this purpose, PRP was obtained from blood samples anticoagulated with either SC or ACD; platelets were lysed; coagulation was activated or not by CaCl_2_ addition and supernatants (containing the platelet releasates, PRPr) were used as inducers of HMEC-1 proliferation and migration, both responses required during angiogenesis. Platelet poor plasma (PPP) was used as control. In contrast with the isolated and attached morphology of cells observed before treatments, incubation of HMEC-1 for 24 h with PPP or PRPr without addition of CaCl_2_ resulted in the aggrupation of cells in clusters and no cell proliferation (Figure 1A). Normal cobblestone endothelial cell morphology was observed by treatment of HMEC-1 after calcium restitution confirming the crucial role of this cation in cellular adhesion and morphology. Moreover, cell proliferation from the original number of 15 × 10^3^cells (dotted line) was triggered by PRPr, being that induced by SC-PRPr higher than ACD- (Figure 1A). Similar to the proliferation results, cell migration was not observed in cells exposed to PPP although the angiogenic response was triggered by SC-PRPr but not ACD- (Figure 1B). Moreover, alteration of cellular morphology evidenced as disruption and density changes of confluent monolayer limiting wounds was observed after experimental conditions without CaCl_2_. Analysis of cellular viability showed that 15 and 20% of HMEC-1 death was detected after 24 h of incubation with SC-PRPr and ACD- without CaCl_2_, respectively (Table 1). Levels of cellular death were reduced after restitution of CaCl_2_ without showing significant differences between SC-PRPr and ACD- treatments (Table 1). These results suggest that decrease of angiogenic responses observed in non-calcium conditions could be partially explained by endothelial death. In contrast, differences in cell proliferation and migration between SC-PRPr and ACD- were not associated to a differential induction of cellular death (Table 1) neither to a differential recovery in platelet number or levels of growth factors on PRPr (VEGF, PDGF, EGF, bFGF, and TGFβ) (Table 2). To rule out that results were associated with the use of an immortalized cell line, the experiments were repeated using a primary cell culture of HUVEC. Proliferation and migration of endothelial cells was again only induced after calcium restitution, being that induced by SC-PRPr higher than ACD- (Figures 1C,D). Similar results were also observed in the treatments containing a commercial growth factors-enriched medium (EGM2) instead PRPr indicating that the effect exerted by anticoagulants is extended to different types of endothelial cells and culture media. In addition, a similar pattern-effect exerted by anticoagulants was observed on HMEC-1 and HUVEC viability. Specifically, while 22–28% of HUVEC death was found after 24 h of incubation with non-calcium treatments, cellular viability was significantly increase after CaCl_2_ addition (Table 1). After restitution of calcium, not significant differences on viability were observed between SC-PRPr and -ACD. Therefore, and similar with proliferation and migration findings, the effect of anticoagulants is extended to different types of endothelial cells.

**TABLE 1:** HMEC-1 and HUVEC viability.

Cellular viability after 24 h of PRPr (%)	HMEC-1		HUVEC	
		
	-CaCl_2_	+CaCl_2_	−CaCl_2_	+CaCl_2_
PRPr-SC	85 ± 8	97 ± 6*	78 ± 8	93 ± 7*
PRPr-ACD	80 ± 7	89 ± 6*	72 ± 8	86 ± 7*
PRPr-SC + Glucose	–	91 ± 8	–	90 ± 8
PRPr-1/2SC	–	97 ± 6	–	94 ± 6
PRPr-1/2ACD	–	95 ± 7	–	91 ± 8
*n* = 4, *P < 0.05 vs. -CaCl_2_				

**TABLE 2:** Hematology parameters and growth factors levels.

	SC	ACD	1/2SC	1/2ACD
**Whole Blood (WB)**				
WBC (×10^9^/L)	7.60.6	7.70.7	7.50.5	7.80.7
RBC (×10^12^/L)	4.60.2	4.50.3	4.80.3	4.80.2
Plt (×10^9^/L)	24348	22732	26247	23941
**Platelet Rich Plasma (PRP)**				
WBC (×10^9^/L)	0.00.0	0.00.0	0.00.0	0.00.0
RBC (×10^12^/L)	0.00.0	0.00.0	0.00.0	0.00.0
Plt (×10^9^/L)	43568	502106	49084	45178
Plt (fold of WB)	2	2	2	2
Plt (% recovery)	625	6310	668	6310
pH	7.50.6	7.40.6	7.60.7	7.60.8
**Levels of Growth Factors in lysed PRP releasates (PRPr)**				
VEGF (pg/ml)	11750958	10900656	12417740	11233525
PDGF (pg/ml)	290611985	276081924	332221685	324451598
EGF (pg/ml)	27742	27235	30639	31058
bFGF (pg/ml)	35322	38015	36830	36626
TGFβ (pg/ml)	2008198	2063175	1866162	2032178

*Blood and PRP samples were examined using a hematology analyzer (Abacus Junior^®^). The percentage of platelet recovery was calculated as (Platelet concentration in PRP × volume PRP/Platelet concentration in WB × volume WB) × 100. Levels of growth factors in releasates of lysed PRP (PRPr) were determined by ELISA.*

### ACD but Not SC Induces Inflammation and Impairs PRPr-Mediated Regeneration *in vivo*

**FIGURE 2:**
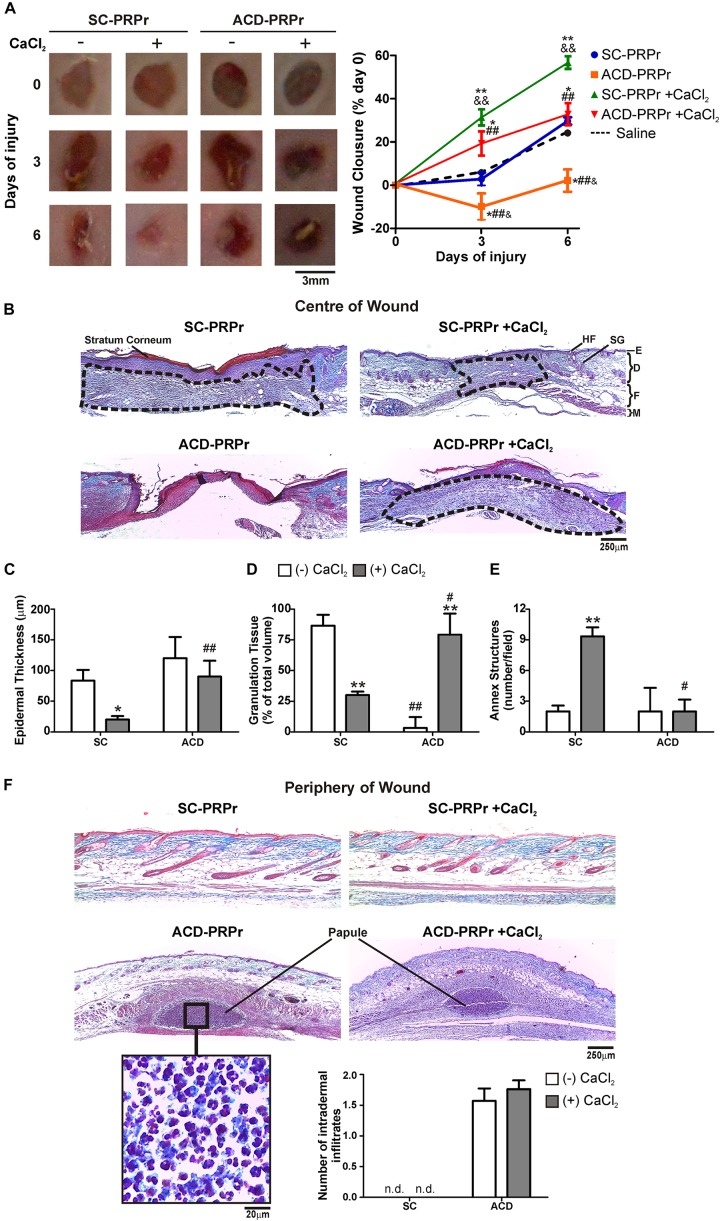
ACD but not SC induces inflammation and impairs PRPr-mediated mouse skin regeneration. Four round full-thickness excisional wounds of 3 mm in diameter were generated in the back skin of female BALB/c mice (8–10 weeks old). SC-PRPr or ACD- obtained from other mice were supplemented or not with CaCl_2_ and injected subcutaneously in the periphery of wounds. Saline was used as control. Healing was analyzed at 3 and 6 days post-injury. **(A)** Wounds were photographed and wound closure% was determined by quantification of wounds perimeters using the ImageJ software. (*n* = 14; ^&^*P* < 0.05, ^&&^*P* < 0.01 vs. saline; *P < 0.05; **P < 0.01 vs. CaCl_2_ (-); ^##^*P* < 0.01 vs. SC-PRPr. Repeated Measures One-way ANOVA followed by Fisher test). **(B)** Skin biopsies obtained on day 6 were stained with Masson’s trichrome. Images of the center of wounds were captured using an inverted microscope. **(C)** Epidermal thickness, **(D)** granulation tissue volume (dotted lines), and **(E)** annex structures (hair follicles and sebaceous glands) were quantified using the ImageJ software. **(F)** Images of the periphery of wounds were captured and intradermal inflammatory infiltrates were quantified. SG, sebaceous gland; HF, hair follicle; E, epidermis; D, dermis; F, fat layer; M, muscle layer. (Magnification 100X). (*n* = 14; *P < 0.05; **P < 0.01 vs. CaCl_2_ (-); ^#^*P* < 0.05, ^##^*P* < 0.01 vs. SC-PRPr. Two-way ANOVA followed by Fisher test).

To study the relevance of the *in vitro* findings we next analyzed the effect of citrate-anticoagulants in a model of skin regeneration *in vivo* (Etulain et al., 2018). For these experiments, PRP was obtained from murine blood collected with SC or ACD. After lysis of platelets, coagulation was activated or not with CaCl_2_ and the four different treatments (1:SC; 2:SC + CaCl_2_; 3:ACD; 4:ACD + CaCl_2_) were subcutaneously injected in the periphery of four round full-thickness excisional wounds generated in the back skin of other mice. Wound healing was monitored at 3 and 6 days post-injury. Our findings indicate that the kinetic of macroscopic healing mediated by SC-PRPr was accelerated by restitution of calcium levels. Specifically, while wound closure was not modified after 3 days by PRPr-SC without calcium, 31 ± 6% closure was reached in the presence of calcium (Figure 2A). These differences were also evidenced at day 6 reaching 57 ± 6% of wound closure by PRPr-SC + CaCl_2_, significantly higher than the 25 ± 3 and 22 ± 2% induced by PRPr-SC (-CaCl_2_) or saline, respectively. On the other hand, wound perimeter was increased after 3 days of treatment with ACD-PRPr (-CaCl_2_) resulting in a negative value in the wound closure% and suggesting that tissue damage was induced by this experimental condition. Similar results were observed when ACD was injected with saline instead of PRPr (Supplementary Figure 1) indicating that ACD injection exerts a direct harmful effect on mouse skin. Even when the harmful effect of ACD was not observed when calcium was restored, the wound closure after 6 days of ACD-PRPr did not exceed the physiologic wound closure of the saline control group (Figure 2A, dotted line) indicating that the regenerative capacities of PRP are inhibited by ACD regardless the presence of calcium.

To further understand the effect of anticoagulants on the regenerative properties of PRP, the histological examination of skin biopsies stained with Masson’s trichrome was performed on day 6 (Figure 2B). Three different histological criteria were used to evaluate wound healing: (1) epidermal regeneration quantified as a reduction in hyperplastic neoepidermis thickness (Figure 2C); (2) granulation tissue development as a provisional matrix for regeneration (Figure 2D); and (3) regeneration of skin mature annex structures, including sebaceous glands (SG) and hair follicles (HF) (Figure 2E; Etulain et al., 2018). Images obtained in the center of wounds revealed that after 6 days of treatment with SC-PRPr (without calcium) the neoepidermis remained thick and hyperplastic due to the presence of stratum corneum (red) and a thick papillary dermis (adjacent below stratum corneum) (Figures 2B,C). Development of large granulation tissue (dotted line) constituted by fibroblasts and dermal matrix collagen (blue) was also evidenced under these conditions (Figures 2B,D). Similar results were obtained with saline controls (Supplementary Figure 1) indicating that regeneration was not accelerated in this PRPr condition. In contrast, a significant reduction in epidermal thickness and granulation tissue volume together with the incipient regeneration of annex structures was observed after SC-PRPr + CaCl_2_ treatment (Figures 2B–E). Regarding ACD, a delay on granulation tissue formation was observed in wounds treated with ACD-PRPr –CaCl_2_. Although it was normalized after calcium restitution, this treatment was not able to induce peripheral dermal regeneration as it was induced by the equivalent condition obtained with SC.

Together with the analysis performed in the center of wounds, the presence of dermal inflammatory infiltrates (papules) containing leukocytes was evidenced in the periphery of wounds treated with PRPr containing ACD ± CaCl_2_ (Figure 2F). This phenomenon was not associated with the presence of leukocytes in PRP (not shown). In addition, the inflammatory papules were not observed in the periphery of wounds treated with PRPr containing SC (Figure 2F) or ACD-Saline (Supplementary Figure 1F). These results indicate that an adverse inflammatory reaction is exerted by the combination of ACD and PRPr.

### The D-Glucose (Dextrose) Is Responsible for the Anti-Angiogenic, Anti-Regenerative and Inflammatory Effect Exerted by ACD

**FIGURE 3:**
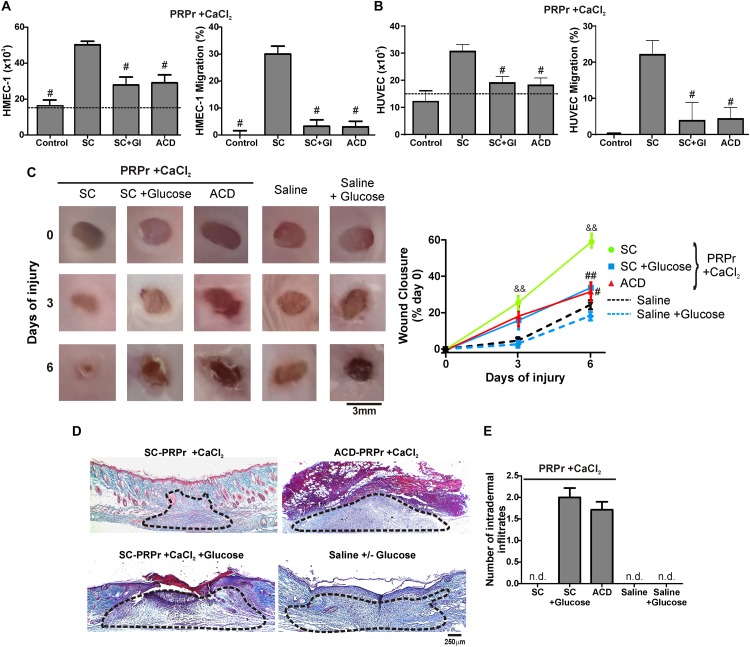
D-Glucose (Dextrose) confers anti-angiogenic, anti-regenerative and inflammatory proprieties to PRP. HMEC-1 **(A)** or HUVEC **(B)** were incubated with SC-PRPr + CaCl_2_ (supplemented or not with Glucose 18 mM) or ACD-PRPr + CaCl_2_. MCDB-131 medium **(A)** or Endothelial Basal Medium 2 (EBM2) **(B)** both supplemented with FBS 2% were used as control. Endothelial cell proliferation at 24 h and migration at 8 h were determined (*n* = 4; ^#^*P* < 0.05 vs. SC-PRPr. Kruskal-Wallis test followed by Dunn test). **(C–E)** Murine SC-PRPr + CaCl_2_ (supplemented or not with Glucose 18 mM) or ACD- were injected subcutaneously in the periphery of wounds generated in back skin of other mice. Saline supplemented or not with Glucose was used as control. Healing was analyzed at 3 and 6 days post-injury. **(C)** Wounds were photographed to determine the wound closure% (*n* = 8, ^&&^*P* < 0.01 vs. saline; ^#^*P* < 0.05, ^##^*P* < 0.01 vs. SC-PRPr. Repeated Measures One-way ANOVA followed by Fisher test). **(D)** Skin biopsies obtained on day 6 were stained with Masson’s trichrome. Images of the center of wounds were captured using an inverted microscope (Magnification 100X). **(E)** Number of intradermal inflammatory infiltrates in the periphery of wounds was quantified (*n* = 8).

Having shown that the differences between SC and ACD were not reversed after calcium restitution we wonder whether individual components of ACD might be responsible for the angiogenic, regenerative and inflammatory alterations. To evaluate the differences between ACD and SC we focused on dextrose (D-Glucose). For these experiments, SC-PRPr + CaCl_2_ was supplemented or not with 18 mM of glucose as the equivalent concentration of dextrose present in PRP that derives from ACD; and it was used to induce angiogenic and regenerative responses. As it is shown in Figure 3, while PRPr obtained with SC was able to induce HMEC-1 and HUVEC proliferation and migration, as well as *in vivo* wound closure and dermal regeneration, all these responses were inhibited in the presence of glucose (Figures 3A–D). The anti-angiogenic and anti-regenerative effects of glucose were not be associated with an increase in the death of endothelial cells since cellular viability after incubation of HMEC-1 and HUVEC with SC-PRPr and SC-PRPr + Glucose was similar (Table 1). Like the effects of ACD-PRPr, the presence of inflammatory infiltrates was observed in the periphery of wounds treated with SC-PRPr + Glucose. In contrast, these papules were not detected in wounds treated with saline controls supplemented or not with glucose indicated that the observed inflammatory effect is exclusively due to the combination of PRPr and glucose (Figure 3E).

### Half Doses of Anticoagulants Increases the Regenerative Capacity of PRP

**FIGURE 4:**
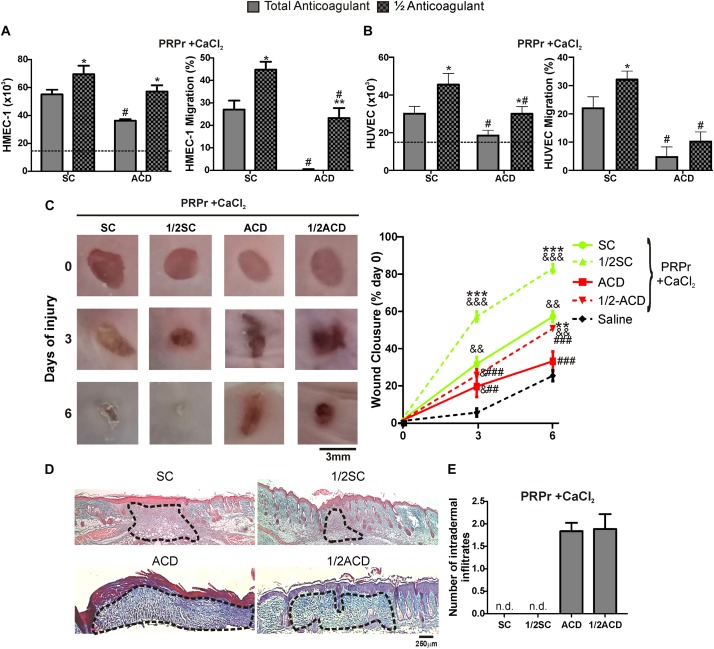
Reduction of SC and ACD concentrations improves the angiogenic and regenerative responses mediated by PRP. HMEC-1 **(A)** or HUVEC **(B)** were incubated with PRPr obtained with total or half (1/2) concentration of SC or ACD. Endothelial cell proliferation at 24 h and migration at 8 h were determined (*n* = 4–5; *P < 0.05, **P < 0.01 vs. total anticoagulant; ^#^*P* < 0.05 vs. SC-PRPr. Two-way ANOVA followed by Fisher test). **(C)** Murine PRP was obtained with total or half (1/2) concentration of SC or ACD. Platelets were lysed, coagulation was induced by CaCl_2_ and PRPr were obtained and injected subcutaneously in the periphery of wounds generated in back skin of other mice. Saline was used as control. Healing was analyzed at 3 and 6 days post-injury. Wounds were photographed to determine the wound closure% (*n* = 8. ^&^*P* < 0.05, ^&&^*P* < 0.01, ^&&&^*P* < 0.001 vs. saline; **P < 0.01, ***P < 0.001 vs. total anticoagulant; ^##^*P* < 0.01, ^###^*P* < 0.001 vs. SC-PRPr. Repeated Measures One-way ANOVA followed by Fisher test). **(D)** Skin biopsies obtained on day 6 were stained with Masson’s trichrome. Images of the center of wounds were captured using an inverted microscope (Magnification 100X). **(E)** Number of intradermal inflammatory infiltrates in the periphery of wounds was quantified (*n* = 8).

A recent study showed that proliferation of fibroblasts induced after supplementation of basal medium with 20% of PRP increases when SC is reduced by half (Anitua et al., 2016). In order to extend these results to our model, blood was collected with total or half (1/2) concentrations of SC or ACD. Our findings show that both proliferation and migration were significantly increased by reduction of anticoagulant concentration (Figures 4A,B). Angiogenic differences between anticoagulant concentrations were not associated to differences in the number of platelets recovered in PRP, neither in pH or growth factor levels (Table 2). Regarding *in vivo* experiments, the anti-regenerative and pro-inflammatory effect of ACD-PRPr was slightly reduced and still present in the treatments containing half ACD (Figures 4C–E). In contrast, a significant improvement on the regenerative capacity of PRP was observed by reduction of SC, reaching an 82 ± 7% of wound closure and almost complete dermal regeneration after 6 days of treatment.

## Discussion

Although PRP is used as a source of platelet-derived growth factors in regenerative medicine, its effectiveness remains controversial partially due to the absence of PRP preparation protocols based on the regenerative role of platelets (Etulain et al., 2018). Instead, the current preparation methods are focused on the hemostatic functions of platelets for transfusion purposes. Unlike these protocols where platelets containing anticoagulants are administered intravenously, during regenerative protocols platelets (and their additives) are administered locally in damaged tissues which are often not vascularized. Thus, it is conceivable that the elimination of anticoagulant during these local administrations differs from those described for systemic administration. Our hypothesis is that anticoagulants exert a harmful and local effect that interferes with the regenerative proprieties of platelets. To test this hypothesis, we compared the effect of SC and ACD on the overall angiogenic and regenerative responses targeted by platelets.

Through *in vitro* studies we first demonstrated that the calcium-chelating effect exerted by SC and ACD alters the morphology of endothelial cells resulting in the inhibition of endothelial proliferation and migration mediated by platelet releasates. These results agree with the fundamental concepts about the role of calcium-signaling pathways in cellular biology. Calcium is a second messenger essential for the homeostasis of all cellular and tissue processes and it triggers the activation of several metabolic steps including cellular death (Moccia et al., 2012; Dubois et al., 2016; Bootman et al., 2018; Paudel et al., 2018). We also found that endothelial viability was reduced in the absence of CaCl_2_ suggesting that decrease of angiogenic responses observed in non-calcium conditions could be partially explained by endothelial death. After restitution of calcium, proliferation and migration of endothelial cells were normalized when PRP was obtained with SC but not with ACD. In these conditions, diminution on endothelial viability by anticoagulated-PRPr or significant differences between SC-PRPr and ACD- were not observed. Thus, differences between treatments at physiologic levels of calcium would not be explained by a cytotoxic effect of anticoagulants. Other possibilities to explain this anti-angiogenic phenomenon could be attributable to a cytostatic effect exerted by anticoagulants. In addition, a metabolic effect of citrate independent of its anticoagulant proprieties cannot be rule out. It could include the alteration of intracellular pH, acid-based homeostasis and intracellular citrate metabolism traditionally demonstrated in the context of urine metabolism (Hamm, 1990).

Intriguingly, the anti-angiogenic effect of ACD has not been reported in previous studies that evaluated proliferation of other cells induced by PRPr *in vitro* (Lei et al., 2009; do Amaral et al., 2016; Zhang et al., 2019). Of note, and in contrast with our experimental settings where cells were incubated with medium containing 100% of PRPr, the previous reports were performed using complete culture medium supplemented with 1–20% of PRPr suggesting that the effect of anticoagulants could have been bypassed by dilution. Indeed, we have recently demonstrated that the dilution of PRPr with saline between 25 and 50% increases the angiogenic and regenerative responses mediated by platelets. This phenomenon was explained by the optimal balance reached after dilution between pro- and anti-angiogenic factors derived from platelets and plasma, respectively (Etulain et al., 2018). Now we propose that another benefit of using diluted PRPr is the consequent reduction of anticoagulants additive in the solution.

In agreement with the *in vitro* results, our *in vivo* findings indicated both, that calcium supplementation is crucial to restore the regenerative ability of anticoagulated-PRP and that wound healing is delayed by ACD regardless the presence of calcium. In addition, the presence of intradermal inflammatory infiltrates was observed in the periphery of wounds treated with ACD-PRPr. These anti-regenerative and pro-inflammatory responses observed with ACD-PRPr were reproduced when SC-PRPr was supplemented with glucose (18 mM) indicating that glucose derived from ACD interferes with regeneration mediated by platelets. Accordingly, and although normal levels of glucose (5.5 mM) are essential for cellular metabolism, it is well known that intermedia or high levels of this molecule (10–30 mM) exert anti-angiogenic, inflammatory and cytotoxic effects that contributes to the development of chronic wounds in patients with diabetes (Shakya et al., 2015; Hu and Lan, 2016). Intriguingly, a delay in wound healing or the presence of inflammatory papules were not observed in mice injected with glucose alone (saline + glucose controls) indicating that these phenomena are not induced by glucose alone but in combination with PRPr. In the same line of evidence, an *in vitro* study has demonstrated that glucose alone (15–25 mM) failed to induce cellular activation processes required for the infiltration of neutrophils into inflamed tissue as chemotaxis and adhesion. However, in combination with the chemoattractant stimulus fMLP, both inflammatory cellular responses were synergistically increased in the presence of glucose (Oldenborg and Sehlin, 1999). In addition, a previous work published by our group has demonstrated that inhibition of *in vitro* angiogenic responses (including endothelial proliferation, migration, survival and tubule formation) induced by TNF-α is increased in combination with glucose (Mena et al., 2018). These results suggest that glucose synergizes with other stimuli to enhance pro-inflammatory and anti-angiogenic responses. It is well known that platelet alpha granules not only contain growth factors but other molecules including cytokines and chemokines (e.g., SDF-1α, IL-8, IL-17, TNF-α, RANTES, ENA-78, MCP-1) (Etulain et al., 2012, 2018; Amable et al., 2013; Andia and Abate, 2013; Anitua et al., 2019). Thus, it is conceivable that local cytokines derived from platelets act in combination with glucose to induce the recruitment of inflammatory cells into the dermis, and to inhibit the angiogenesis required during wound healing and tissue regeneration. It is also conceivable that local presence of high glucose levels induces the activation and cytokine production from tissue and immune cells as it is traditionally known in the context of diabetes (Donath et al., 2019). The requirement of glucose in anticoagulants and preservative solutions have been stablished to avoid lysis of erythrocytes during storage of these cells for transfusion (Mollison, 2000). Considering that red cells are not required for using PRP for regenerative purposes, the addition of glucose could be avoided, and SC could be used as anticoagulant instead of ACD.

Finally, and according with a recent publication about the effect of diluting SC to increase fibroblast proliferation mediated by PRPr (Anitua et al., 2016), we found that half reduction of SC and ACD resulted in the improvement of endothelial proliferation and migration mediated by PRPr. In contrast, platelet-mediated regenerative responses were only improved by reduction of SC but not ACD indicating that anti-regenerative effect exerted by this anticoagulant is not prevented by dilution. This phenomenon might be associated with the persistence of inflammatory infiltrates in 1/2ACD-PRP impeding the resolution of inflammation required during wound healing (Zhao et al., 2016; Sorg et al., 2017). In agreement with our data, two recent studies have showed that PRP obtained from anticoagulated blood is better to induce proliferative and regenerative responses mediated by platelets (Miron et al., 2017; Du et al., 2018). One of these studies has demonstrated that mouse skin wound closure is faster induced by PRPr prepared without anticoagulant than ACD-PRPr (Du et al., 2018). Controversially, the development of intradermal inflammatory papules after ACD-PRPr injection was not mentioned in this study. Of note, it is unknown whether the inflammatory phenomenon that we have observed using Balb/c mice could be also developed in Nude mice that have an alteration of immune system, not only in T-cells but also a defective activation of granulocytes and monocytes (Wysoczynski et al., 2017). Besides these discrepancies, we agree that using non-coagulated blood is a physiological alternative to reduce the harmful effect of anticoagulants. However, spontaneous coagulation of blood obtained without anticoagulant constitutes a limitation in handling times from blood extraction to PRP application. Thus, reduction of anticoagulant could be an alternative balance to reduce the anti-regenerative effect of anticoagulants allowing convenient manipulation. Indeed, and in agreement with Anitua findings (Anitua et al., 2016), in preliminary settings we have observed that basal levels of hemostatic functions of PRP (including aggregation and coagulation) were similar and completely inactivated between total and half concentration of anticoagulants-PRP. In agreement again with Anitua (Anitua et al., 2016), activation responses induced by stimulus addition (as PAR-1 activated peptide) were similar induced in the presence of both concentrations of anticoagulants, but they were reached 20–40% faster in treatments containing half amount of SC or ACD (data not shown). Thus, hemostatic proprieties of PRP are conserved in treatments containing half amount of anticoagulants.

Extrapolation of our findings to the clinic might explain the inflammatory reactions observed in patients after receiving regenerative and local treatments with PRP (Bowman et al., 2013; Kaux et al., 2014; Alam et al., 2018; Latalski et al., 2019). These complications were not associated with the presence of leukocytes in PRP since all the interventions were performed using free-leukocyte PRP. Of note, all of these procedures were performed using PRP anticoagulated with ACD and without calcium restoration arguing about a possible toxic effect caused by the anticoagulant. Unfortunately, and in contrast with the industrialized medical drugs that require pharmacovigilance, the heterogeneity of platelet-derived products hinders the establishment of regulatory frameworks worldwide required to apply a mandatory and unified record of side effects (Anitua et al., 2015; Beitzel et al., 2015). To count with more of these registers would allow a deeper understanding about the adverse effects of anticoagulants used in regenerative protocols mediated by platelets allowing providing therapeutic strategies to prevent and treat these complications.

## Conclussion

Our findings demonstrate that reduction of calcium levels exerted by citrate-anticoagulants interferes with the angiogenic and regenerative proprieties of platelets. This effect is reversed by restitution of calcium levels. D-Glucose (Dextrose) contained in ACD confers anti-angiogenic, anti-regenerative and inflammatory proprieties to PRP suggesting that, in contrast with protocols for obtaining blood-derivatives for transfusion, SC could be considered for obtaining platelets for regeneration. Finally, blood obtained with half amount of SC is the best alternative to improve the regenerative responses mediated by PRP.

## Data Availability Statement

All datasets generated for this study are included in the article/Supplementary Material.

## Ethics Statement

The studies involving human participants were reviewed and approved by the Ethic Committee of the National Academy of Medicine (T.I. 12910/18/X). The patients/participants provided their written informed consent to participate in this study. The animal study was reviewed and approved by the Institutional Committee for Care and Use of Laboratory Animals (CICUAL 059/2018). IMEX-CONICET/National Academy of Medicine.

## Author Contributions

PO performed the experiments and analyzed the data. PZ assisted in the performing experiments. MS assisted with design of the work objectives, acquired funding, and provided a critical and substantive review of manuscript. JE conceived and designed the work, performed experiments, analyzed data, acquired funding, wrote the manuscript and approved the final version of work. All authors reviewed the manuscript.

## Conflict of Interest

The authors declare that the research was conducted in the absence of any commercial or financial relationships that could be construed as a potential conflict of interest.
