# Supplementary material for: Anticoagulants Interfere With the Angiogenic and Regenerative Responses Mediated by Platelets
Source: Front Bioeng Biotechnol. 2020 Mar 20;8:223. doi: 10.3389/fbioe.2020.00223 (PMC7098916; doi:10.3389/fbioe.2020.00223)
Supplement: Supplementary file 1 [file Data_Sheet_1.PDF]

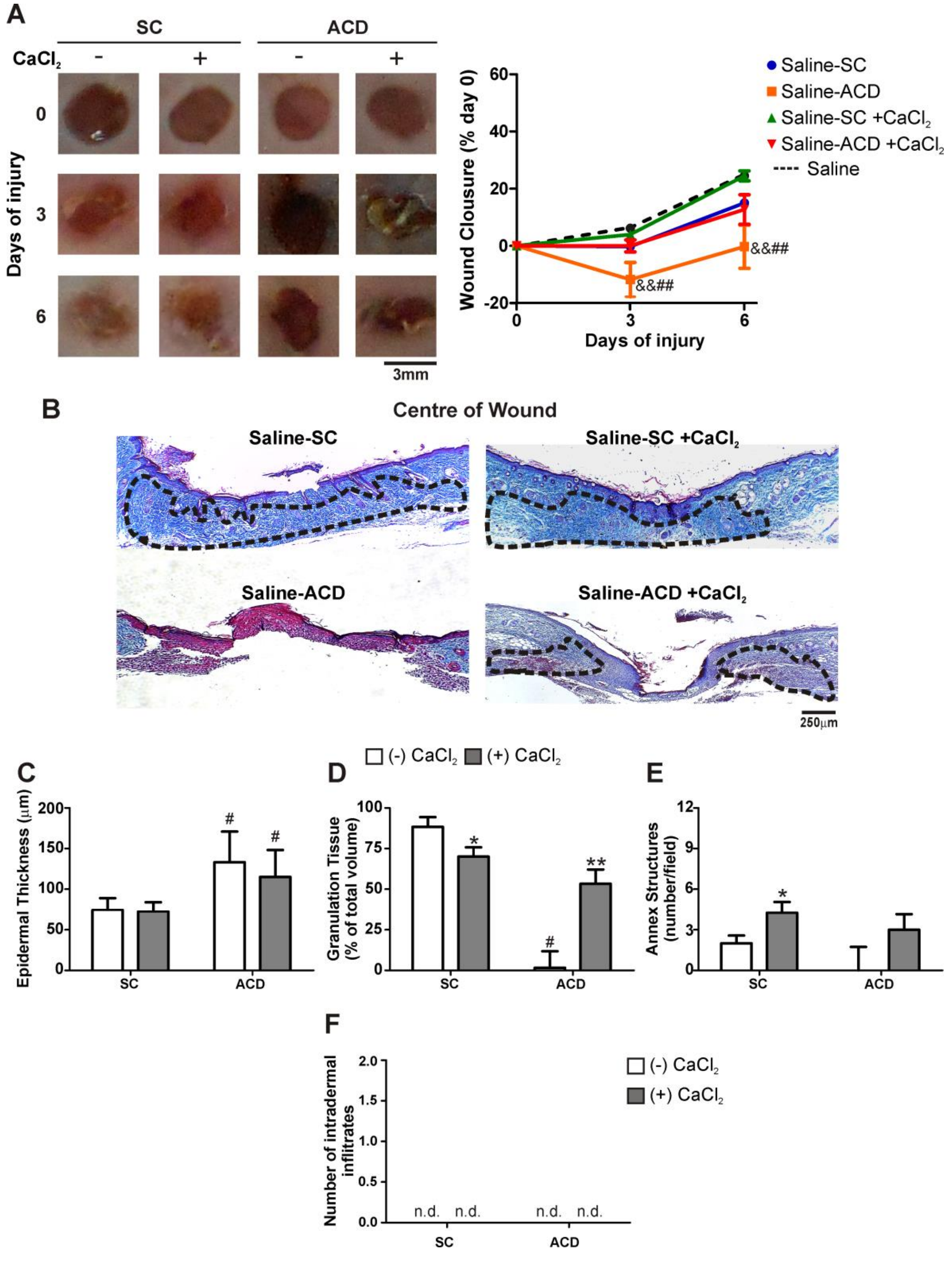

Supplemental Figure 1. Effect of saline supplemented with ACD and SC in mouse skin regeneration. Saline containing SC or ACD was supplemented or not with $CaCl_2$ and injected subcutaneously in the periphery of full-thickness excisional wounds generated in the back skin of

female BALB/c mice (8-10 weeks old). A) Wounds were photographed and wound closure % was determined by quantification of wounds perimeters using the ImageJ software. (n=6; &&P<0.01 vs saline; ##P<0.01 vs Saline-SC. Repeated Measures One-way ANOVA followed by Fisher test). B) Skin biopsies obtained on day 6 were stained with Masson's trichrome. Images of the centre of wounds were captured using an inverted microscope. C) Epidermal thickness, D) granulation tissue volume (dotted lines), and E) annex structures (hair follicles and sebaceous glands) were quantified using the ImageJ software. F) Presence of intradermal inflammatory infiltrates in the periphery of wounds was not detected (n.d.). (Magnification 100X). (n=6; *P<0.05, **P<0.01 vs $CaCl_2$ (-); #P<0.05 vs. Saline-SC. Two-way ANOVA followed by Fisher test).
